# Low-Programmable-Voltage Nonvolatile Memory Devices Based on Omega-shaped Gate Organic Ferroelectric P(VDF-TrFE) Field Effect Transistors Using p-type Silicon Nanowire Channels

**DOI:** 10.1007/s40820-014-0016-2

**Published:** 2014-10-23

**Authors:** Ngoc Huynh Van, Jae-Hyun Lee, Dongmok Whang, Dae Joon Kang

**Affiliations:** 1 Department of Physics, Institute of Basic Sciences, Sungkyunkwan University, Suwon, 440-746 Republic of Korea; 2 School of Advanced Materials Science and Engineering, SKKU Advanced Institute of Nanotechnology, Sungkyunkwan University, Suwon, 440-746 Republic of Korea

**Keywords:** Si nanowires, Field effect transistor, Ferroelectric memory

## Abstract

A facile approach was demonstrated for fabricating high-performance nonvolatile memory devices based on ferroelectric-gate field effect transistors using a p-type Si nanowire coated with omega-shaped gate organic ferroelectric poly(vinylidene fluoride-trifluoroethylene) (P(VDF-TrFE)). We overcame the interfacial layer problem by incorporating P(VDF-TrFE) as a ferroelectric gate using a low-temperature fabrication process. Our memory devices exhibited excellent memory characteristics with a low programming voltage of ±5 V, a large modulation in channel conductance between ON and OFF states exceeding 10^5^, a long retention time greater than 3 × 10^4^ s, and a high endurance of over 10^5^ programming cycles while maintaining an *I*_ON_/*I*_OFF_ ratio higher than 10^2^.

## Introduction

Ferroelectric-gate field effect transistor (FeFET)-based memory has many potential applications owing to its unique properties, including fast programming and erase speed, low power consumption, small cell size, low-voltage operation, high rewriting endurance, and non-volatility. In particular, building ferroelectric memory with low power consumption and low operation voltage is critical for energy-saving and long-term operation of modern potable electronic devices. However, researchers face significant challenges, such as depolarization fields and charge trapping, which degrade the device performance, thereby preventing ferroelectric memory from realizing its potential [1–5]. One-dimensional (1D) nanostructures such as nanowires (NWs), nanotubes, and nanocables have attracted much attention over the past decades as conducting channels for FeFETs owing to their unique physical properties [6]. Many reports have suggested that nanowire ferroelectric field effect transistors (NW FeFETs) have higher performance, that is, longer retention time, and better endurance memory characteristics compared with thin-film-based FeFET memory devices. For example, an NW with small dimensions is desirable for increasing the area density of devices, and a single-crystalline semiconductor NW can function as a superior carrier transport channel with enhanced field effect mobility (*μ*_*eff*_), subthreshold slope (SS), and operation voltage [7, 8]. The superior mobility and small size of CNTs and semiconducting 2-dimensional graphene ribbons have also been exploited to surpass the latest silicon (Si) technology in device operation speed [9, 10]. Despite great progress in the development of these materials, graphene FeFETs still suffer from a low *I*_ON_/*I*_OFF_ ratio and the OFF-state leakage problem owing to various undesirable tunneling mechanisms and ambipolar behavior. Furthermore, the difficulty in selecting metallic or semiconductor CNTs continues to limit the industrial applications of these materials. For the foregoing reasons, Si is considered the best candidate for a material for conducting channels, with regard to mobility [7, 8].

Ferroelectric memory devices constructed using Si as a semiconducting channel generally experience difficulties in practical applications due to problems of interface diffusion and the formation of natural silicon oxide at the interface. Therefore, to obtain a good interface between ferroelectric oxide materials such as lead zirconate titanate (PZT) or bismuth titanium oxide (BTO) on Si semiconducting channels, a buffer layer between them is necessary, which can increase the depolarization field and working voltage [1, 4]. Organic ferroelectric materials for nonvolatile memory devices have been exploited by many research groups because of their easy fabrication, light weight, flexibility, solution-based large area application, and low-cost fabrication. In addition, using organic ferroelectric materials, adding a buffer layer is not required, because of their low-temperature processing capability [2].

In this work, we demonstrated that the interfacial layer problem with a Si NW FeFET can be remedied by incorporating poly(vinylidene fluoride-trifluoroethylene) P(VDF-TrFE) as an organic ferroelectric gate into p-type Si NW to form FeFET nonvolatile memory devices. The omega-shaped gate allows for the enhancement of device performance and extremely low power consumption of memory devices when using p-type Si NWs as a conducting channel. We optimized the doping concentration such that our FeFET memory devices exhibit excellent memory characteristics with a low programming voltage while maintaining a large modulation in channel conductance and a long retention time. This memory device architecture is promising for next-generation nonvolatile memory for flexible substrate applications.

## Experimental

The boron-doped p-type Si NWs used in this study were synthesized using a nanocluster-catalyzed vapor-liquid-solid transport method in a low-pressure chemical vapor deposition reactor with a silane (SiH_4_) to diborane (B_2_H_6_) gas ratio of 5,500:1. This synthesis procedure is described in detail elsewhere [11]. Single-crystal Si NWs with a typical diameter of 50–70 nm were first dispersed by ultrasonication in isopropanol and then transferred onto a substrate by dropping a liquid suspension of Si NWs using a pipette. A heavily doped p-type Si substrate was employed as a back gate with a 100 nm thick thermal oxide layer on top as a gate oxide layer. Source and drain electrodes were patterned by photolithography followed by electron-beam evaporation of 80 nm Ni and 50 nm Au electrode on a Si NW to form Ohmic contact between NWs and electrodes, along with a lift-off process. Before the electrode deposition, Si NWs were etched in a 1 % hydrofluoric acid solution for 15 s to remove a native oxide layer on the shell of the NWs [11]. The 200 nm thick P(VDF-TrFE) ferroelectric layer was prepared by mixing P(VDF-TrFE) at 0.08 wt% in 2-butanone. The solution was then coated on the Si nanowire field effect transistors (NW FETs) by spin coating (1 min at 1,000 rpm), which had been previously treated in a 2 min oxygen plasma treatment, thermal annealing (2 h at 80 °C and 2 h at 130 °C in air) was performed to form a β-phase dominant P(VDF-TrFE) [12]. The electrical characteristics of the devices were measured in air using a probe station with a Keithley SCS-4200 system.

## Results and Discussion

**Fig. 1:**
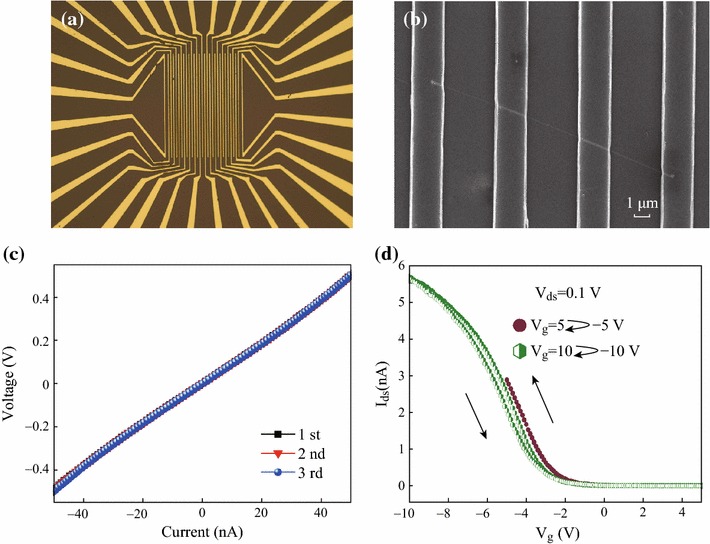
**a** Optical and **b** FE-SEM images of p-type Si NW FET devices; **c** four probe measurements and **d** hysteresis behaviors for a back-gate Si NW FET during the gate voltage scan from +5 to −5 V, +10 to −10 V, and back

To investigate the basic electrical properties of p-type Si NWs, we prepared a conventional NW FET on a 100 nm SiO_2_/Si substrate with a back gate. Figures 1a, b are the optical and field-emission scanning electron microscopy (FE-SEM) images of p-type Si NW FET devices. The Si NW resistance R = 10^7^ Ω was extrapolated from the linear region of the current–voltage curve of four probe measurements (Fig. 1c). The resistivity *ρ* = 0.48 Ω cm was calculated according to *ρ* = *RA*/*L*, where *A* = π*r*^2^ is the Si NW cross-section, *L* is the conducting channel length of the nanowire (~5 µm), and *r* is the radius of the nanowire (~27.5 nm).The small hysteresis windows were observed in the conventional back-gate Si NW FET during the gate voltage scan from +5 to −5 V; +10 to −10 V; and back, exhibiting the typical electrical behavior of a p-type channel Si NW FET (Fig. 1d). This hysteresis was caused by the trapped charges at the SiO_2_/Si interface due to immobile oxide charges or oxide-trapped space charges associated with defects at the SiO_2_ surface [13].

**Fig. 2:**
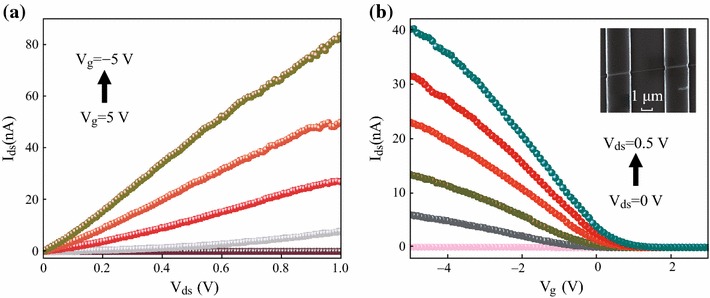
Electrical transport properties of single Si NW FET device in ambient conditions; **a***I*_*ds*_ − *V*_*ds*_ output characteristics and **b***I*_*ds*_ − *V*_*g*_ transfer characteristics

The curves in Fig. 2a depict the measured drain current versus the drain-source voltage (*I*_*ds*_ − *V*_*ds*_) for a single Si NW FET. The conductance of the NW increases monotonically as the gate potential decreases in the range from +5 to −5 V, demonstrating a p-channel Si NW FET. The curves in Fig. 2b show the drain current versus the gate-source voltage (*I*_*ds*_ − *V*_*g*_) by sweeping the gate voltage from 5 to −10 V with a drain voltage from 0 to 0.5 V. The transconductance (*g*_*m*_) and the field effect hole mobility (*µ*_*h*_) can be determined using the *I*_*ds*_ − *V*_*g*_ curves according to the following equations: $g_{m}=dI_{ds}/dV_{g}$ and *μ*_*h*_ = *g*_*m*_*L*^2^/*C*_*ox*_*V*_*ds*_, respectively, for the back-gate NW FETs [5]. *C*_*ox*_ is the gate oxide capacitance of a cylindrical wire on a planar substrate and can be calculated as *C*_*ox*_ = 2*πɛ*_*r*_*ɛ*_0_*L*/ cosh^−1^(1 + *t*_*ox*_/*r*), where ε_r_ = 3.9 is the relative dielectric constant, *t*_*ox*_ = 100 nm is the thickness of the SiO_2_ gate dielectric layer, respectively. For NW FETs on a SiO_2_/Si substrate, the threshold voltage *V*_*th*_ = 1 V and transconductance *g*_*m*_ = 4.2 nS were extrapolated from the linear region of the *I*_*ds*_ − *V*_*g*_ curve at *V*_*ds*_ = 0.1 V. The field effect hole mobility was determined as *µ*_*h*_ = 21.4 cm^2^/V s. The hole carrier concentration *n*_*h*_ was calculated as *n*_*h*_ = *1/ρqμ*_*h*_ = 6.2 × 10^17^/cm^3^. The subthreshold swing $S=\log\;[\text{d}V_{g}/\text{d}\left(\log I_{ds}\right)]$ is about was determined as 205 mV/dec, which is considered small for low power consumption devices.

**Fig. 3:**
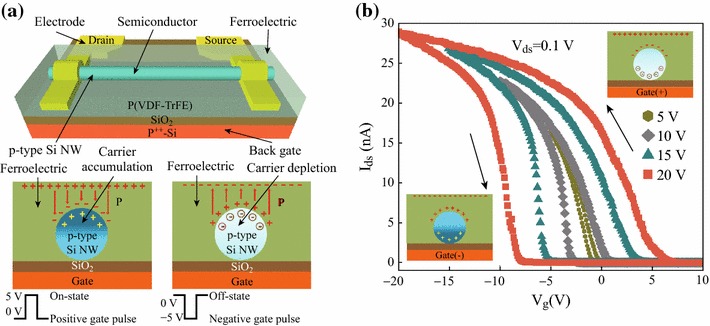
Si NW FeFET memory device. **a** Schematic view and operating mechanism of a back-gate FeFET-based nonvolatile memory device. **b** Hysteresis behavior for a back-gate Si NW FeFET-coated P(VDF-TrFE) on the NW surface during the gate voltage scan from +5 to −5 V, +10 to −10 V, +15 to −15 V, +20 to −20 V, and back

A schematic of the back-gate FeFET-based nonvolatile memory device and the operation mechanism of the Si NW FeFET is shown in Fig. 3a. The FeFET operating mechanism was proposed by Liao et al. [5]. In our device structure, rather than using the ferroelectric layer as the back-gate dielectric layer, we coated a ferroelectric P(VDF-TrFE) on the top of the Si NW.

Hysteresis curves of a back-gate Si NW FeFET with respect to the sweep range of gate voltages are shown in Fig. 3b. Positive or negative polarization of P(VDF-TrFE) is induced on a Si NW depending on the gate voltage sweep direction, causing modulation in the channel conductance and threshold voltages. In our device, a ~ 1 V counter-clockwise hysteresis loop window was observed with conductance changes of more than five orders of magnitude at high and low conductance states when sweeping the back-gate voltage from +5 to −5 V and back to +5 V, with a sweeping step of 0.1 V at *V*_*ds*_ = 0.1 V in an ambient environment. Additionally, a hysteresis loop window of up to ~ 12 V was observed when the back-gate voltage was swept from +20 V to −20 V and back to +20 V. The hysteresis window becomes larger at a higher gate voltage sweeping range, confirming that such hysteresis behavior is due to the polarization of the ferroelectric layer.

**Fig. 4:**
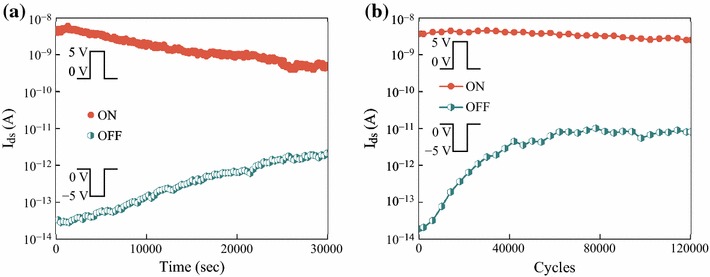
Memory properties of a Si NW-coated P(VDF-TrFE) FeFET-based device: **a** retention times and **b** endurance tests of the drain currents of the Si NW FeFET device. The gate pulse was ±5 V with 100 ms pulse width and *V*_*ds*_ = 0.1 V

The memory properties of the P(VDF-TrFE)-coated Si NW FeFET-based memory are evidenced by retention times (Fig. 4a) and endurance tests (Fig. 4b) for the high and low conductance states, *i.e.*, the ON and OFF states of a Si NW FeFET memory device. These were measured at *V*_*ds*_ = 0.1 V and *V*_*g*_ = 0 V after the device was switched ON and OFF using +5 V writing and −5 V erasing pulses, respectively, with pulse widths of 100 ms. A large change in conductance between ON and OFF states, exceeding 10^5^, was obtained and remained over 10^2^ even after 3 × 10^4^ s (~8 h). The OFF current of our memory device is ~ 10^−14^ A, nearly 10 times lower than the lowest *I*_OFF_ reported by Fang et al. [14] for NW FET devices and 100 times lower than values for bulk CMOS. The ON current, <5 nA, is the lowest *I*_ON_ reported with a high *I*_ON_/*I*_OFF_ exceeding 10^5^. At *V*_*ds*_ = 0.1 V, the power dissipation of the ON state was measured to be as low as ≤ 0.5 nW. To the best of our knowledge, this demonstrates lower ON-state power dissipation and programming voltage operation than any nanowires, CNTs, or graphene-based 1D conducting channel FeFET memory counterparts [9, 10, 13, 15–17] that maintain a sufficiency high *I*_ON_/*I*_OFF_ ratio and long retention time. Our memory devices support operation at extremely low power compared with other NW FeFET memory and bulk CMOS Ferroelectric memory counterparts. Furthermore, a high endurance of over 10^5^ programming cycles was observed with an *I*_ON_/*I*_OFF_ ratio greater than 10^2^ (Fig. 4b). The endurance test for both ON and OFF states was conducted after 4,000 programming cycles with *V*_*ds*_ fixed as 0.1 V, a cycle condition of ±5 V peak-to-peak pulse, and a 100 ms pulse width.

The long retention time and high endurance of Si NW FeFET memory, which are related to the ferroelectric-gate NW FET memory structure and Si/ferroelectric interface, are explained as follows. The depolarization field and carrier charge trapping are the main factors underlying the short retention time of FeFET-based memory [1, 2]. According to Ma et al. [1], the depolarization field is dependent on remnant polarization *P*_*r*_. For a NW FeFET, the remnant polarization of the ferroelectric material should be sufficiently large to produce an electric field that induces the OFF state on the conducting channel of the NW FET. That is, the remnant polarization must produce an electric field stronger than the gate electric field that induces the threshold voltage of the NW FET. The novel feature of our approach is achieving a long memory retention time by adjusting the threshold voltage of the Si NW FET. Lower threshold voltage and smaller remnant polarization not only reduces the depolarization field but also minimizes the leakage current and trapped carrier charges at the interface between the conducting channel and the ferroelectric layer. One effective way to tune the threshold voltage is to reduce the doping concentrations as demonstrated in our previous work [18]. With smaller remnant polarization, a thinner ferroelectric layer and lower programming voltage is required. We aim to achieve a low programming voltage while maintaining a sufficiency high *I*_ON_/*I*_OFF_ ratio. Such change in the doping concentrations was realized by varying gas ratios of silane (SiH_4_) to diborane (B_2_H_6_). When these ratios were varied from 3,000:1 to 6,500:1, the positive threshold voltage shifted from +5 V to approximately 0 V as doping concentration was reduced. Additionally, the reduction of ON current from 10^−7^ to 10^−9^ A was observed when the hole carrier concentrations were lowered with decreasing doping concentrations. Reducing the doping concentration leads to fewer hole carriers in the conducting p-type channel Si NWs, resulting in reduced conductance and a negative shift of the threshold voltage of the Si NW FETs. Furthermore, further reduction in threshold voltage also deteriorates the performance (conductivity, transconductance, mobility and the *I*_ON_*/I*_OFF_ ratio) of the FET [18]. To maintain a sufficient *I*_ON_*/I*_OFF_ ratio exceeding 10^5^ and to achieve long retention time memory devices, the doping concentration was optimized at a 5,500:1 gas ratio of SiH_4_:B_2_H_6_, and threshold voltage of ~ 1 V.

The electrical properties of Si NWs are highly dependent on the dimension and the surface states. The surface-defects-induced p-type conduction of Si NWs was reported by Luo et al. [19]. For n-type Si NW FETs, p-type behavior becomes dominant in the negative gate voltage region owing to surface defects, and *I*_OFF_ becomes higher with lower gate voltage. This reduces the *I*_ON_/*I*_OFF_ ratio of n-type Si NW FETs, and therefore, the p-type Si NW FET has a higher *I*_ON_/*I*_OFF_ ratio than the n-type Si NW FET. For memory applications, lower operation gate voltage to reduce the depolarization field for a longer retention time of Si NW FeFET is expected [1]. This leads to a reduction in threshold voltage and conductivity of NW FETs. Again, the *I*_ON_/*I*_OFF_ ratio of n-type Si NW FETs will gradually be reduced. Thus, p-type Si NWs are preferable for obtaining long retention time, low operation voltage, and low power consumption of memory devices while maintaining a reasonable *I*_ON_/*I*_OFF_ ratio. Although the mobility of p-type Si NWs is inferior compared with that of n-type Si NWs, p-type Si NWs are considered the most suitable compared with metal oxide semiconductors.

The advantage of NW as a semiconducting channel in FeFET memory devices is the sensitive changes in conductance and the inherently small scale of each device, leading to a high memory device density. Single-crystalline nanowires and nanotubes often feature greater mobility and lower swing, allowing FET devices to operate at higher speed and lower power consumption. Because NW FETs function in accumulation and depletion modes, they allow lower operation voltage than thin-film FETs operating in inversion mode. Lower operation voltage in FeFETs can reduce the depolarization field in the ferroelectric layer and the charge trapping induced at the semiconducting/ferroelectric interface, leading to a longer memory state retention time [1, 2]. Furthermore, to form an omega-shaped-gate FeFET structure [20], the Si NW was coated with P(VDF-TrFE). Without a gate dielectric layer, the polarization of the ferroelectric layer will affect directly on the surface of the NW. This provides a more effective electric field with respect to the NW FET than the omega-shaped-gate and planar-gate FETs. FeFET devices are also expected to exhibit a lower operation voltage, higher field effect mobility, higher transconductance, and higher *I*_ON_/*I*_OFF_ ratio than the other devices [20].

It is extremely difficult to deposit high-quality ferroelectric metal oxides such as PZT and BTO on Si substrates with a good interface because of the chemical reaction and inter-diffusion of Si into the ferroelectric layer [21]. Such effects create a gate leakage current and trapping carriers in the gate dielectric stack, resulting in reduced retention time of the memory devices [1]. To reduce the leakage current and charge tapping, a thick buffer layer at the interface, comprising SiO_2_, Al_2_O_3_, or HfO_2_, is employed for thin-film-based FeFET memory. However, adding the buffer layer causes other problems: the data retention period becomes shorter, and thus, higher gate voltages are required owing to the effect of the depolarization field [1–4]. In our memory devices, P(VDF-TrFE) was used as a ferroelectric gate. The ferroelectricity can be easily induced by a thermal annealing after spin coating above the Curie temperature (but lower than 140 °C), results in no interfacial layer formation during the crystallization process. Without an interfacial layer, the charge trapping effect at the interface between the organic film surface and the Si NW semiconductor channel can be minimized, making a buffer layer unnecessary. When direct contact is realized in a FeFET, the data retention time is likely to be far longer than that in a conventional FeFET because no depolarization field is generated, owing to the buffer layer. Moreover, with a low-temperature fabrication process, these devices can be fabricated on flexible substrates for flexible nonvolatile memory applications. Compared with the BTO particle-coated ZnO NW FeFET memory devices reported by Sohn et al. [15], our devices exhibit a more uniform coating and allow facile control of the thickness of the ferroelectric layer by simply changing the spin-coating speed.

**Fig. 5:**
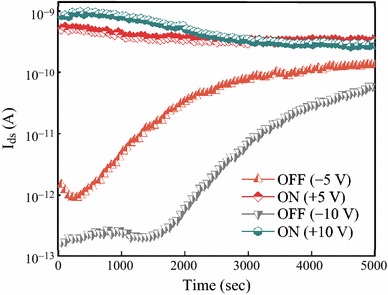
Retention times for the ON and OFF states of Si NW FET measured at *V*_*ds*_ = 0.1 V and *V*_*g*_ = 0 V after the device was switched ON and OFF using +5; +10 V writing and −5; −10 V erasing pulses; the pulse width was 100 ms

We also investigated the memory properties of Si NW FET without P(VDF-TrFE) coated. Retention times for the ON and OFF states, caused by the effects of trapped charges at the SiO_2_/Si interface [13], of Si NW FET measured after the device was switched ON and OFF using (Fig. 5a) +5 V writing and −5 V erasing pulses and (Fig. 5b) +10 V writing and −10 V erasing pulses. A rapid reduction in the conductance between the ON and OFF states to over 10^3^ after 1.5 × 10^3^ s (less than 0.5 h) was observed after a device was switched ON and OFF using +10 V writing and −10 V erasing pulses, respectively. This is another confirmation that the measured memory properties of P(VDF-TrFE)-coated Si NW FeFET were due to the remnant polarization of the ferroelectric layer.

## Conclusions

In summary, FeFET using p-type Si NW and P(VDF-TrFE) ferroelectric omega-shaped gates were employed successfully to fabricate memory devices. The device was carefully characterized with respect to the electrical transport, retention, and endurance time. A long retention time, high endurance, and high *I*_ON_/*I*_OFF_ ratio were obtained even with a 1 V hysteresis window and ±5 V writing and erasing gate pulses. Extremely low power dissipation of the ON state, as low as ≤0.5 nW, was also observed. These results offer a viable method to fabricate high-performance, high-density nonvolatile memory devices that is suitable for flexible electronics because it involves a low-temperature processing technique.
